# Giant mesenteric cyst: Successful management in low-resource setting

**DOI:** 10.1016/j.ijscr.2020.04.070

**Published:** 2020-05-07

**Authors:** Mario Antunes, Damiano Pizzol, Marcella Schiavone, Anna Claudia Colangelo

**Affiliations:** a Department of Surgery, Central Hospital of Beira, Beira, Mozambique; b Operational Research Unit, Doctors with Africa Cuamm, Mozambique; c Department of Emergency and Organ Transplantation-Section of Thoracic Surgery, University of Bari, Bari, Italy; d Department of Surgery and Organ Transplantation, University of Padua, Padua, Italy

**Keywords:** Giant mesenteric cyst, Lymphatic cyst, Mesothelial cyst, Low-income setting

## Abstract

**Introduction:**

Mesenteric cysts are rare, generally benign intra-abdominal lesions with a wide range of presentation in terms of size, clinical presentation, etiology, radiological features, and pathological characteristics.

**Presentation of case:**

We reported a case of giant mesenteric cyst in a 16-month-old girl successfully managed in a low-resource setting.

**Discussion:**

This case is particularly important not only due to the rarity of the presented case, but also for the highlighted aspects from a public health point of view.

We faced of the problem of a late stage disease and the lack of preoperative diagnosis due to cultural and economic reasons and the weaknesses of healthcare systems, as in the majority of low- and middle-income countries.

**Conclusion:**

Despite all these limitation, this case illustrates that complex, rare diseases can also be managed successfully in a low-resource setting. It is mandatory to strengthen and improve the health system both in terms of equipment both in terms of public health policies in order to offer a better and more effective quality of care to patients also in low-income countries.

## Highlights

• Mesenteric cysts are rare lesions with a wide range of aetiology, clinical presentation and patholo-gical characteristics.
• The preoperative diagnosis is done by ultrasound and computerised tomography to determine extension and cystic content.
• The gold standard treatment is the operative therapy also to exclude malignant transformation and to prevent complications.
• The management in low-income countries is difficult due to the lack of human and materials re-sources.

## 1 Introduction

Mesenteric cysts are rare, generally benign intra-abdominal lesions with an incidence ranging from 1 in 105,000 to 1 in 250,000 among admitted surgical patients [1]. Mesenteric cysts have a wide range of presentation in terms of size, clinical presentation, etiology, radiological features, and pathological characteristics [2]. In fact, the average size ranges from 2 to 35 cm and, thus, patients present with nonspecific complaints of abdominal pain, distension, or an abdominal mass [2]. Although several classifications have been proposed, the most widely recognized includes 4 groups based on clinical and etiological features: 1) lymphatic, 2) mesothelial, 3) enteric, 4) urogenital, 5) mature cystic teratoma and 6) non pancreatic pseudocysts [3]. In general, simple lymphatic and mesothelial cysts are usually asymptomatic, while lymphangiomas and benign cystic mesotheliomas can be aggressive and invasive [3]. Finally, the only malignant is the malignant cystic mesothelioma [3]. Simple lymphatic and mesothelial cysts seems to have a congenital etiology, while lymphangiomas and benign cystic mesotheliomas have not a known origin [3]. The diagnosis is mainly based on clinical and radiological findings with histological confirmation [3]. Surgical removal is considered the gold standard procedure especially for giant cysts and minimally invasive surgery is the approach of choice [4].

We reported a case of giant mesenteric cyst successfully managed in a low-resource setting. The work has been reported in line with the SCARE criteria [5].

## 2 Ethic statement

Written informed consent was obtained from the parents of the girl for publication of this case report and any accompanying images.

## 3 Case report

**Fig. 1:**
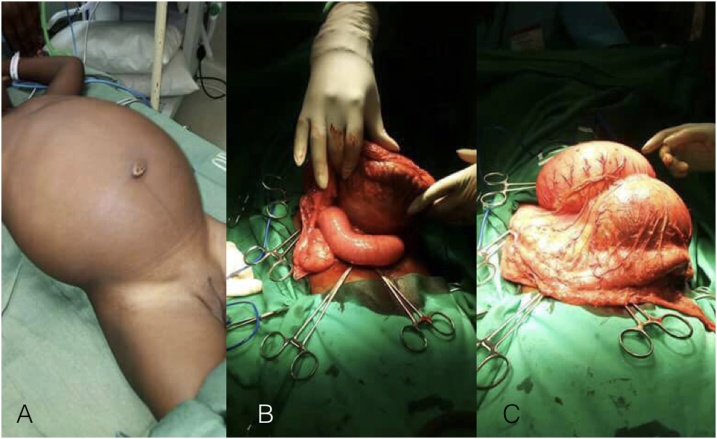
Giant mesenteric cyst at presentation (A) and intra-operative images (B and C).

A 16-month-old girl presented with diffuse abdominal distention, nausea, vomiting and severe pain after one month history of abdominal discomfort. The physical examination, made difficult by painful palpation, showed severe abdominal distention with a palpable, soft and fluid-filled mass (Fig. 1A). Plain abdominal radiographs showed air–fluid levels and distended bowel loops, suggesting intestinal occlusion. Without the possibility to perform more adequate imaging tests, and worsening the conditions of the girl, we planned a surgical intervention. We performed a laparotomy and found a giant, soft, cystic, milky fluid–filled mass in the mesentery of the ileum (Fig. 1B and C). The intestinal resection of about 10 cm of the involved loops was necessary. Pathological examination confirmed a diagnosis of mesenteric cystic lymphangioma containing chylous milky fluid. We removed a mass containing about 400 ml fluid.The following postoperative course was regular. On the tenth postoperative day the girl was discharged.

## 4 Discussion

This is a very rare case considering that lymphangiomas incidence ranges from 1 in 105,000 to 1 in 250,000 among admitted surgical patients and are predominant in male [1]. Mesenteric cysts are frequently symptomatic and localized in the mesentery but can appear also in adults and middle aged women mainly in retroperitoneal space [6,7]. The clinical presentation is influenced by the size of the cyst and the age of patients [7]. During childhood, it can simulate an appendicitis, while in adults is often asymptomatic [7,8]. The symptoms are generally a-specific and include pain, nausea and vomiting, constipation or diarrhea. Moreover, an abdominal palpable mass is present in up to 61% of the patients [9].

Usually, the preoperative diagnosis is done by ultrasound in order to distinguish between plain and cystic masses and computerized tomography (CT) or magnetic resonance imaging (MRI) to determine extension and cystic content [10]. Needle aspiration or explorative laparoscopy can further help in differentiating between a pancreatic pseudocyst, a benign cyst mesothelioma or a lymphangioma before any operative procedure [10].

Unfortunately, in our case we had no adequate diagnostic imaging and we preferred to directly choose the surgery option considering the late stage situation with noticeable symptoms. The surgical treatment is mandatory for large mesenteric cysts in order to exclude malignant transformation and to prevent complications, such as rupture, hemorrhage, torsion or infections. In case of simple lymphatic and mesothelial cysts, it is easy and curative to enucleate the mass, but lymphangiomas and benign cystic mesotheliomas could be adherent to vital intra-abdominal structures and their complete excision could be very difficult or impossible. Although limphangiomas and benign cystic mesothelioma are considered benign tumors and, thus, should be avoided the resection of vital organs, in some cases is reported a resection of different organs due to adhesion [6,7]. In our case, we performed a 10 cm of intestinal resection without remove any part of terminal ileum. This is a crucial aspect for a good grow and development of the girl considering that she shouldn't have important consequences in terms of nutrients absorption and, thus, nutritional status.

Recurrence and metastasis are not frequent and occur especially if the resection is incomplete [11].

We were able to confirm the hypnotized diagnosis first of all by macroscopic characteristics that are useful for the distinction from simple lymphatic cysts (small and unilocular), to lymphangiomas (large and multi-loculated, with multiple cysts). Moreover, we confirmed the diagnosis by the histological examination characterizing endothelial and mesothelial cells.

This case is particularly important not only due to the rarity of the presented case, but also for the highlighted aspects from a public health point of view. In fact, as the result of the weaknesses of healthcare systems and also due to cultural and economic reasons, as in the majority of low- and middle-income countries we faced of the problem of a preoperative diagnosis lacking ultrasound, CT and MRI scanners. Moreover, although it is not the case, surgical and anesthesiology equipment is often poor being impossible to perform a complete preoperative or laparoscopic diagnostic procedure. In this case, an explorative laparotomy was necessary without a clear preoperative diagnosis, but this would probably not have changed outcome. In a high-resource country, a laparoscopic approach would have been used to perform the whole operation that we made by open surgery. Despite all these limitation, this case illustrates that complex, rare diseases can also be managed successfully in a low-resource setting. Unfortunately, we lost the patient in follow up due to difficult access to the hospital, living far from the hospital and due to the lack of an effective network and system for monitoring and ensuring follow-up visits.

Therefore, it is mandatory to strengthen and improve the health system both in terms of equipment both in terms of public health policies in order to offer a better and more effective quality of care to patients also in low-income countries.

## Conflict of interest

All authors declare no conflict of interest

## Funding

No funds was received for this work

## Ethical approval

Written informed consent was obtained from the parents of the child for publication of this case report and any accompanying images.

Moreover the ethical approval has been exempted by our institution considering that the case was written using retrospective and anonymous data.

## Consent

Written informed consent was obtained from the parents of the child for publication of this case report and any accompanying images.

## Author contribution

MA: data collection and interpretation and final revision.

DP: writing paper and data interpretation.

MS: writing paper.

ACC: data analysis and final revision.

## Registration of research studies

This is a case report and it is not registered.

## Guarantor

Mario Antunes.

## Provenance and peer review

Not commissioned, externally peer-reviewed.
